# An Investigation into Hydraulic Permeability of Fibrous Membranes with Nonwoven Random and Quasi-Parallel Structures

**DOI:** 10.3390/membranes12010054

**Published:** 2021-12-31

**Authors:** Zeman Liu, Yiqi Wang, Fei Guo

**Affiliations:** 1 Key Laboratory of Ocean Energy Utilization and Energy Conservation of Ministry of Education, Dalian 116024, China; coolee444@gmail.com (Z.L.); wangyiqi@dlut.edu.cn (Y.W.); 2 School of Energy and Power Engineering, Dalian University of Technology, Dalian 116024, China; 3 School of Mechanical Engineering, Dalian University of Technology, Dalian 116024, China

**Keywords:** hydraulic permeability, compressibility, quasi-parallel, nonwoven, electrospinning

## Abstract

Fibrous membranes with a nonwoven random structure and a quasi-parallel fibrous structure can be fabricated by the electrospinning technique. The membranes with different structures exhibited different behaviors to a hydraulic flow passing through the membranes. This work presents the effects of the fiber arrangement, fiber diameter, and deformations of the fibers on the hydraulic permeability. The results showed that the hydraulic flow can generate an extrusion pressure which affects the porosity and pore structure of the fibrous membranes. The quasi-parallel fibrous membranes and nonwoven membranes exhibited similar variation tendencies to the change of the experimental variables. However, the quasi-parallel fibrous membranes exhibited a higher sensibility to the change of the hydraulic flow rate. The hydraulic permeability of the quasi-parallel fibrous membranes was further analyzed with packing state models in this work.

## 1. Introduction

Electrospun fibrous membranes are considered as the next generation of membranes for liquid filtration processes [1,2,3] and air filtration processes [4,5], due to their extremely high surface-to-volume ratios and high porosity [6], which lead to a high separation efficiency and high permeability [7,8]. Electrospun membranes are usually compressible, which certainly affects their permeability [9]. It is important to understand the mechanism of the compressibility and permeability of electrospun membranes to find out their potential applications along with other kinds of membranes.

The compressibility of the fibrous medium under pressure was first studied by Van Wyk [10]. In his work, nonrecoverable deformations were neglected for simplicity. Since then, more complex models have been proposed by Carnaby and Pan [11,12], Komori et al. [13,14], Toll [15], and Baudequin et.al. [16], in which more factors were considered. The compression process of a fibrous medium can be described by Jönsson and Jönsson [17]. Several mechanisms contribute to this process, which are deformations of fibers, bending and slippage of fibers, and disintegration of fiber groups. 

The permeability of a fibrous medium depends on the size, concentration, and arrangement of fibers [18]. There are several studies on the permeability of porous fibrous media. Models to determine the permeability constant, when the hydraulic flow goes, through the aligned cylinders that are parallel [19,20] or perpendicular [19,20,21] to the flow direction were proposed. The effect of the fiber orientation was presented by Mao and Russel [22,23]. Numerical methods were used to propose equations describing the permeability of the membrane [21,24,25]. Although Happel’s Method was proposed under the condition of aligned cylinders, it fits the experimental data of nonwoven electrospun membranes as well according to Choong et al. [26]. Recently, the influences of the porosity, pore size distribution, and fiber diameter on the permeability of fibrous media have been studied by using mathematical models [27,28,29] and experiments [30].

The nonwoven structure is a common structure of electrospun fibrous membranes, which are neither woven nor knitted. Fibers are usually without any alignment in a membrane. Quasi-parallel fibrous (QPF) [31] membranes have unique structures in which most of the fibers are aligned in a similar direction. It can be easily fabricated by electrospinning with a relatively higher rolling speed according to the previous work [26]. QPF membranes can be categorized as membranes still with a nonwoven structure, but their general alignment also gives them different properties compared to the totally random structure. There has been a lack of study on the properties of QPF electrospun membranes under hydraulic flow so far. In this work, both nonwoven random and QPF membranes were fabricated and studied. The factors such as the fiber diameter, porosity, and deformations were considered to find out the effects of the fiber arrangement on the permeability and compressibility of the membrane. Some unique properties and applications of the QPF membrane have been proposed.

## 2. Experimental

### 2.1. Materials

Polyacrylonitrile (PAN; molecular weight: 85,000 g/mol; density: 1.184 g/cm3) was obtained from The Dow Chemical Company (Midland, MI, USA). Acetone (>99.5%) was obtained from Tianjin Kemiou Chemical Reagent Co., Ltd. (Tianjin, China). N, N-dimethylformamide (DMF; >99.5%) was obtained from Shanghai Aladdin Bio-Chem Technology Co., LTD. (Shanghai, China). All the materials were used as received without further purification.

### 2.2. Electrospinning

**Figure 1:**
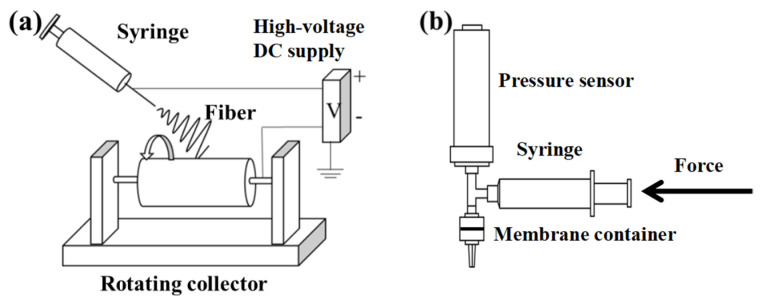
Schematic diagrams of the electrospinning process with a rotating drum collector (**a**) and a hydraulic permeability test unit (**b**).

The polymer was dissolved in a solution containing DMF and acetone at 60 °C with mechanical stirring overnight to prepare PAN solutions with various concentrations. The electrospinning process was not conducted, until the solutions cooled down to room temperature. A rotating drum (diameter: 10 cm) was used to collect fibers (see Figure 1a and Figure S1). Fibrous membranes were used without further modification. The parameters of the electrospinning process are shown in Table 1. The prepared membrane had many advantages, such as the high porosity, controllable fiber diameter, easy fabrication, long-standing structure, and independent use, and can be fabricated into nonwoven random and quasi-parallel structures. However, as shown in Table 1, it is difficult for the QPF membrane with a large fiber diameter (350 nm) to obtain the same thickness as other membranes.

### 2.3. Characterization

#### 2.3.1. Morphology 

A scanning electron microscope (FEI, QUANTA 450, Hillsboro, OR, USA) was used to characterize the morphology of the tested membrane. The pretreatment of the sample membrane was conducted by a sputter coating unit (Quorum Emitech & Polaron Q150T, Laughton, UK) for 90 s to coat a very thin conductive layer of Pt for image enhancement. The SEM was applied to estimate the diameter and the orientation of the fiber as well as the structural changes of the membrane.

#### 2.3.2. Porosity and Fiber Diameter

The permeability of the membrane can be described by Happel’s Method [19]:

(1) $$K=\frac{D^{2}}{32\left(1-\varepsilon \right)}\left(-ln\left(1-\varepsilon \right)+\frac{{\left(1-\varepsilon \right)}^{2}-1}{{\left(1-\varepsilon \right)}^{2}+1}\right),$$

where *K* is the permeability constant, *ε* is the porosity, and *D* is the main fiber diameter of the membrane.

According to Happel’s Method, the permeability of the membrane is affected by its porosity. In this work, the porosity was estimated by the Happel’s Method during each experiment process. The direct measurement, such as the gravimetric method [32], during the test was inconvenient and exhibited experimental error, which may affect the results of the tests.

The fiber diameter also influences the permeability of the membrane. In the electrospinning process, the fiber diameter was mainly determined by the concentration of the PAN solution. It can be measured by software (Nano Measurer 1.2) based on SEM images.

#### 2.3.3. Fiber Orientation

The degree of random and isotropic was indicated by the mean resultant length [33]:

(2) $$\rho =\left(1/n\right)\sqrt{{\left(\overset{n}{\underset{i=1}{\sum }}\cos\theta_{i}\right)}^{2}+{\left(\overset{n}{\underset{i=1}{\sum }}\sin\theta_{i}\right)}^{2}}.$$

As *ρ*→0, the fibers were randomly oriented; as *ρ*→1, the fibers were aligned in a certain direction.

#### 2.3.4. Membrane Thickness

The thickness of the membrane could affect the pressure gradient on the membrane with a given hydraulic flow. It was measured by a digital micrometer (211-101F from Guilin Guanglu Measuring Instrument Co., Ltd, Guilin, China). It was determined by the time length of the electrospinning process.

### 2.4. Hydraulic Permeability and Deformation Test

Darcy’s law can be applied to estimate the hydraulic flow rate across the membrane:

*J* = −(*K*/*μ*)(*dP*/*dz*), (3)

where d*P*/d*z* is the pressure gradient across the membrane, *K* is the permeability constant, *μ* is the dynamic viscosity of water, and *J* is the hydraulic flow rate. 

**Figure 2:**
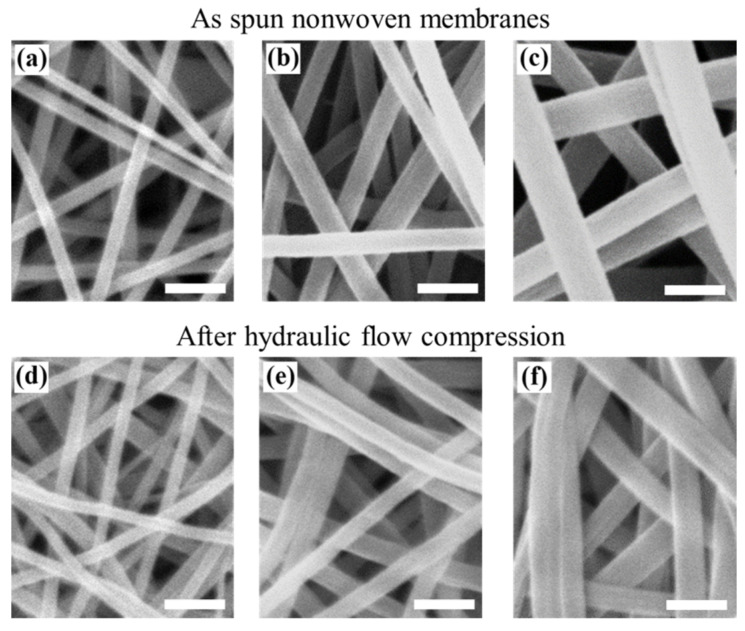
SEM images of electrospun nonwoven PAN membranes with various fiber diameters before the hydraulic tests (**a**–**c**) and after the hydraulic tests (**d**–**f**) (scale bar: 1 μm).

The hydraulic permeability measurement was conducted using a custom-built device (see Figure 2b and Figure S2). The membrane was placed on a substitutable membrane filter made of steel (inner radius *ϕ* = 13 mm). According to Darcy’s law, the pressure is needed to force a hydraulic flow to go through the membrane, even if the membrane is hydrophilic. Deionized (DI) water was used for a hydraulic flow, and the pressure was provided by a syringe pump. The flow rate was controlled by a stepper motor with a range of 0.001–100 mm/min. The pressure measured by a pressure sensor (Beijing Star Sensor Technology CYYZ11-HK-67-RS-16-B-G) was considered as an extrusion pressure generated by the hydraulic flow on the membrane. Experiment data were recorded at 1-s intervals by a computer.

A syringe and a straight tee were filled with water without bubbles left. The tested electrospun membrane was cut into a small round piece with a diameter of 13 mm. Then, it was put into a filter and was wetted subsequently by DI water. The filter was connected to the straight tee. For the hydraulic permeability test, the values of the flow rate ranged from 5 mL/min to 75 mL/min. As shown in Figure S3, during the deformation test, the membrane was under the hydraulic flow compression processes several times with the same flow rate to present the effect of the hydraulic flow on the tested membrane.

## 3. Results and Discussion

### 3.1. Morphology

**Figure 3:**
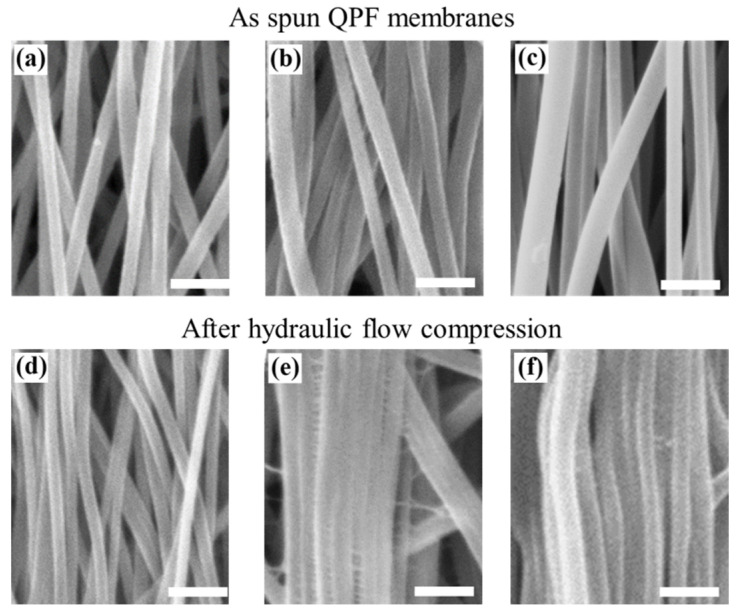
SEM images of electrospun quasi-parallel fibrous (QPF) PAN membranes with various fiber diameters before the hydraulic tests (**a**–**c**) and after the hydraulic tests (**d**–**f**) (scale bar: 1 μm).

Nonwoven membranes (Figure 2) and QPF membranes (Figure 3) were successfully fabricated by electrospinning. The parameters of the membranes can be seen in Table 1. When the drum was at a relatively low rotating speed, the fibers were randomly oriented. When the rotating speed was relatively high, quasi-parallel fibers were obtained. According to Equation (2), the value of *ρ* was calculated to be ~0.96 for all the fibers collected by a drum with a relatively high rotating speed, which meant the fibers were oriented in a certain direction. At the same concentration of the PAN solutions, the fibers in the nonwoven membranes exhibited larger diameters than that in the QPF membranes (see Table 1), due to the relatively higher-speed drum stretching the fibers and making them thinner than those in the nonwoven membranes. Since the fiber with a larger diameter experienced a larger deformation under the same stress–strain behavior, the difference was more obvious for the PAN solution with a higher concentration, which corresponded to a larger fiber diameter. There were mainly point contacts between the fibers in nonwoven membranes, while there were mainly line contacts between the fibers in the QPF membranes. Besides, the line contact would play a more dominant role after the tests for the QPF membranes, while it showed a little increase in the nonwoven membranes. 

### 3.2. Hydraulic Permeability

**Figure 4:**
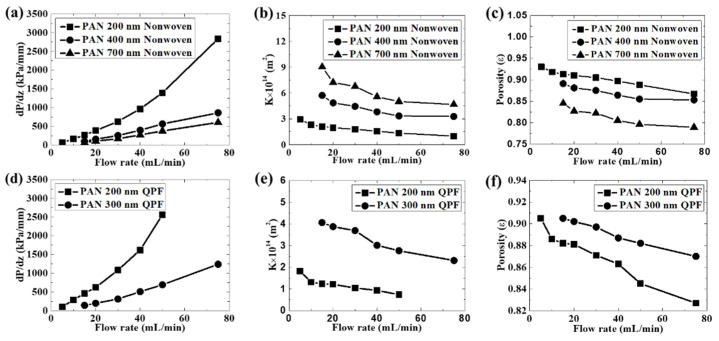
For nonwoven random fibrous membranes with various fiber diameters, the influence of the hydraulic flow on (**a**) hydraulic pressure drop, (**b**) permeability constant, and (**c**) porosity. For QPF membranes with various fiber diameters, the influence of the hydraulic flow on (**d**) hydraulic pressure drop, (**e**) permeability constant, and (**f**) porosity.

**Figure 5:**
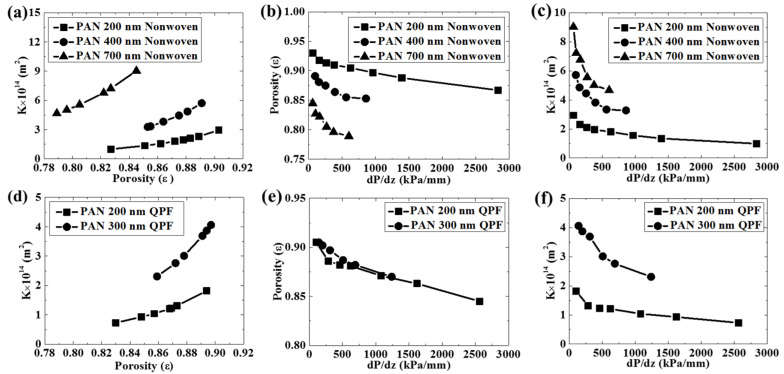
For nonwoven random fibrous membranes with various fiber diameters, the relationships between (**a**) permeability constant and porosity, (**b**) porosity and pressure drop, (**c**) permeability constant and hydraulic pressure drop. For QPF membranes with various fiber diameters, the relationships between (**d**) permeability constant and porosity, (**e**) porosity and pressure drop, (**f**) permeability constant and hydraulic pressure drop.

As shown in Figure 4, the hydraulic flow compression led to a larger pressure drop. When the corresponding pressure according to Darcy’s law was reached, the pressure was nearly constant under relatively lower flow rates. However, it still increased at a lower speed under higher flow rates. This could be due to the compression of the membrane structure under a higher flow rate, which indicated that the membrane was compressible. Inflection points were seen during the experiment. The pressure corresponding to the inflection point was recorded as the extrusion pressure generated by the hydraulic flow during each experiment for simplicity. Actually unrecoverable transformation happened even at a relatively low flow rate during the test. As shown in Figure S3, the hydraulic permeability measurement was conducted at the same flow rate of 10 mL/min for several cycles. It proved that there was unrecoverable transformation, since the extrusion pressure became higher. Besides, the regeneration was also a very important property of the membrane. As shown in Figure S3, the pressure of one curve at the end of the test was higher than the extrusion pressures of the curves beside and above it. However, the extrusion pressure of the curve above was higher than that of the curve below. That meant the membrane was regenerated to some degree. In addition, the relationship was the same between every two adjacent curves, which meant the membrane regenerated partly after each test. However, although we wanted to know the precise radio of the recoverable change and the unrecoverable change of the structure of the membrane, it was difficult to perform the measurement in this test. Both the nonwoven membranes and the QPF membranes exhibited similar variation tendencies to the changes of the experimental parameters. The values of the pressure drop increased along with flow rates, which was in good agreement with Darcy’s law. Moreover, the porosity showed a decrease during the experiment, which meant the membrane was compressible. Both the fiber diameter and the porosity had great influences on the permeability of the membrane. According to Happel’s Method, the permeability constant increased with the increasing porosity and the increasing fiber diameter due to the lower specific area of the contact between the fiber and the hydraulic flow as reported in the literature [9]. As a result, it was easier to let the hydraulic flow go through membranes with larger fiber diameters and higher porosity. As the fiber diameter increased, although the porosity became smaller since fibers with higher diameters led to less void space between them in the membrane, the permeability increased. The fiber diameter played a dominant role in its membrane permeability. The influence of the porosity and the fiber diameter on the permeability is in agreement with the literature [26,27,28,29]. To further investigate the compressibility of the membrane, the relationship between the pressure and the porosity and the permeability of the membrane is shown in Figure 5. According to Happel’s Method, there was a nearly linear relationship between the porosity and the permeability constant K. The porosity and the permeability constant had a similar variation tendency with the pressure. The membrane with a larger fiber diameter was more sensitive to the pressure, which meant it was more compressible. In addition, since the permeability constant of the membrane with a larger diameter was more sensitive to the porosity, it meant the permeability of the membrane with a larger fiber diameter changed more easily under the same pressure condition.

The QPF membrane exhibited some unique properties compared to the nonwoven membrane. As shown in Figure 5, the permeability constant for the QPF membrane was smaller than that of the nonwoven membrane with the same fiber diameter of 200 nm, due to its lower porosity induced by the fiber arrangement. This is in agreement with a former study of Tamayol and Bahrami, which proves that the permeability of the 3D nonwoven structure is larger than that of the 1D parallel structure [21]. The probability of finding an empty reference area is *e−^c^* according to the definition of the Poisson distribution, where *c* is the average surface coverage. When two arrays of cylinders are randomly oriented, the probability is always larger than zero. However, the probability is zero for fibers with a tight parallel structure. This could be explained by considering two arrays of cylinders with the same diameter contacting each other. The arrangements of them are randomly oriented and parallel, respectively. When the cylinders are randomly oriented, there is a void volume between them. When all the cylinders are in the same direction and contact each other side by side, the void volume can be decreased significantly. As a result, the hydraulic flow could generate a higher pressure on QPF membranes than on nonwoven membranes with the same fiber diameter of 200 nm, on which it contacted more fibers and the resistance increased. This could be well described by Happel’s Method and Darcy’s law.

The influence of the fiber diameter on the permeability of the membrane was more obvious in the QPF membranes. The permeability constant increased faster compared to that of the nonwoven membranes, as the fiber diameter increased. Besides, it is noteworthy that the original porosity of the QPF membranes did not decrease with the increasing fiber diameter while it decreased obviously in the nonwoven membranes. It is an abnormal phenomenon, because fibers with larger diameters will cause the lower porosity of the membrane, as mentioned above. One possible explanation is that many fibers in the QPF membranes with a fiber diameter of 300 nm were more likely to adhere to each other because of line contacts between them, which can be observed from SEM images. If there is adhesion between two fibers as mentioned above, they may act as one fiber with a higher fiber diameter. Since the porosity was estimated by Happel’s Method, the larger fiber diameter means the lower porosity under the same permeability constant. The porosity of the membrane with a fiber diameter of 300 nm may be overestimated in this work. This may also explain the rapid increase of the permeability of the QPF membranes since the fiber diameter may be underestimated. Thus, the effect of the fiber adhesion should be taken into consideration in the QPF membranes.

**Figure 6:**
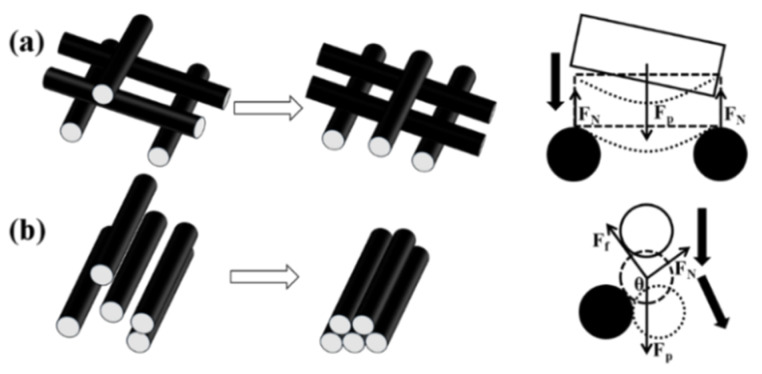
Schematic diagrams of formations of the maximum packing state and force analyses of cylindrical fibers with a criss-cross structure (**a**) and a parallel structure (**b**). The arrows represent the directions of the fiber slippage.

The liquid flow across the membrane leads to the rearrangement of the fibers due to the sliding movement caused by unbalanced hydraulic forces. The fibers tend to form a more compacted structure caused by the hydraulic compression. To further investigate on the mechanisms of the structure variations, a criss-cross structure and a parallel structure were induced to represent the features of the nonwoven membranes and the QPF membranes, respectively. Force analyses were conducted on the fibers with different structures (Figure 6a and Equation (4) for the criss-cross structure and Figure 6b and Equation (5) for the parallel structure):

(4) $$\{\begin{array}{c} F_{n}f=F_{p}\cos\theta \\ F_{n}=F_{p}\sin\theta \end{array},\;$$

*F_n_* = *F_p_*, (5)

where *F_p_* is the force that the hydraulic flow generates on the membrane, *F_n_* is the normal force, *F_f_* is the frictional force, and *f* is the friction factor.

There were mainly point contacts between fibers in the nonwoven membranes. One fiber was surrounded and bolstered by others in all directions and was easy to be stabilized. According to the force analyses, the force conducted by the hydraulic flow was generally along the vertical direction. The slippage between fibers with a criss-cross structure happened at the beginning. Since the fibers overlapped with each other due to the slippage, the fibers were bolstered by those at lower positions. As a result, the amount of slippage was reduced significantly during the compression process, and the membrane will reach its maximum packing state. After that, the fiber exhibited a stress–strain behavior, which caused bending and deformation.

By contrast, the slippage between fibers with parallel structures happened, even if the fiber contacted others below it. There were mainly line contacts in the QPF membranes. The hydraulic force on a single fiber can be resolved into the force normal and parallel to the bottom contacting the fiber. If the component of the hydraulic force parallel to the bottom contacting the fiber was larger than the frictional force, there was still a slippage between fibers. Moreover, the hydraulic flow acted as a lubricator, which certainly contributed to the slippage. The fibers were hard to reach the maximum packing state, until all of them were compressed side by side and the porosity was very low. As a result, the slippage between fibers was more obvious in the QPF membranes. In addition, as mentioned above, the QPF membrane experienced a relatively higher pressure at the same flow rate due to its lower porosity. Figure 6 shows the formation of the maximum packing state for fibers with a criss-cross structure and a parallel structure. There was an inflection point in each experimental curve at around 50 kPa, as shown in Figure 5. Before the inflection point, the slippage played a dominant role, and the porosity changed rapidly. After the infection point, the slippage reduced, which led to a decrease in the change of the porosity, and the structure of the membrane was closer to its maximum packing state.

The changes of the permeability constant and the porosity for the QPF membrane were more obvious than those of the nonwoven membrane with the same fiber diameter of 200 nm during the test, which meant the QPF membrane was more sensible to the flow rate. Figure 5 shows that these two membranes had similar sensitivities to pressure. It meant that the higher pressure generated by the flow on the QPF membrane rather than the difference of the structure change between these two membranes as mentioned above is the main reason for the sensitivity of the QPF membrane to the flow rate under the condition of this test. However, since the porosity of the QPF membrane was lower than that of the nonwoven membrane with the same fiber diameter of 200 nm, it meant the more slippage in the QPF membrane may neutralize the negative effect of the lower porosity on the compressibility of the membrane.

### 3.3. Deformation

The pressure generated by the hydraulic flow on the membrane was icreased after each compression, which resulted from the nonrecoverable deformation. As mentioned above, the contact ways between fibers were quite different in the nonwoven random and QPF structures. The contact area of fibers did not increase much under compression for the nonwoven membranes because of the point contact between fibers. However, the contact area increased due to the slippage of fibers when there were mainly line contacts between fibers. Some integral layers of fibers in the QPF membranes could be seen in the SEM images due to this mechanism, in which fibers contacted each other side by side and looked like an integral layer. This special structure was caused by the slippage between fibers. In the previous study, the nonrecoverable deformations of the fiber itself are often neglected. However, the SEM images indicated the nonrecoverable deformations such as the changes of the shape and diameter of the fiber after compression, which meant that the fiber exhibited stress–strain elastic–plastic behaviors. It is believed that the elasticity of the fiber could affect the compressibility of the membrane.

## 4. Conclusions

PAN solutions with various concentrations can be electrospun into fibrous membranes with nonwoven random and QPF structures. The hydraulic permeability of the fibrous membranes with different structures was studied in this work. The results showed that the hydraulic flow could generate an extrusion pressure, which affected the porosity and the pore structure of the fibrous membranes. Both structures exhibited a similar variation tendency to the change of experimental variables. However, the porosity of the QPF membrane was lower, which caused a lower permeability. The QPF membrane exhibited a higher sensibility to the change of the flow rate. Based on the SEM observation, the fibers were compacted by a hydraulic flow and formed nonrecoverable deformations after liquid penetration. The nonwoven and QPF membranes can be used as common filters in the filtration process. It is also possible to use them as sensors of the flow rate or pressure because of their unique properties. This work provides some preliminary results with a set of proof-of-principle experiments to investigate different performances corresponding to various hydraulic conditions with an ordinary nonwoven fibrous structure and a QPF structure. Further investigations will be proceeded on the behaviors, performance, and potential applications of electrospun fibrous membranes with specific structures.

## Figures and Tables

**Table 1:** Electrospinning processing parameters and the resulting fibrous membrane properties.

Rotating Speed (rpm)	Structure	Flow Rate (mL/min)	Polyacrylonitrile (PAN) Concentration (%)	Fiber Diameter (nm)	Thickness (μm)
40	Nonwoven Random	0.02	11.5 14 16	200 400 700	86 71 96
2800	Quasi Parallel	0.015	11.5 14 16	200 300 350	87 72 40

## Data Availability

Data is contained within the article or Supplementary Materials.

## Supplementary Materials

The following are available online at https://www.mdpi.com/article/10.3390/membranes12010054/s1, Figure S1: The electrospinning system used in this work., Figure S2: A liquid entry pressure (LEP) test unit. A syringe pump was used to generate a pressure on the liquid above the test membrane by pumping the test liquid with a very low but constant flow rate. Figure S3: Schematic diagram of the pressure variation under several cycles of compression at the same flow rate. The pressure increased after each cycle.
