# Supplementary material for: An Investigation into Hydraulic Permeability of Fibrous Membranes with Nonwoven Random and Quasi-Parallel Structures
Source: Membranes (Basel). 2021 Dec 31;12(1):54. doi: 10.3390/membranes12010054 (PMC8780038; doi:10.3390/membranes12010054)
Supplement: Supplementary file 1 [file membranes-12-00054-s001.zip › membranes-1528603-supplementary.pdf]

membranes

Supporting Information

An Investigation Into Hydraulic Permeability of Fibrous Membranes with Nonwoven Random and Quasi-Parallel Structures

Zeman Liu [1,2], Yiqi Wang [1,3] and Fei Guo [1,2,*]

1 Key Laboratory of Ocean Energy Utilization and Energy Conservation of Ministry of Education, Dalian 116024, China; coolee444@gmail.com
2 School of Energy and Power Engineering, Dalian University of Technology, Dalian 116024, China
3 School of Mechanical Engineering, Dalian University of Technology, Dalian 116024, China; wangyiqi@dlut.edu.cn
* Correspondence: feiguo@dlut.edu.cn

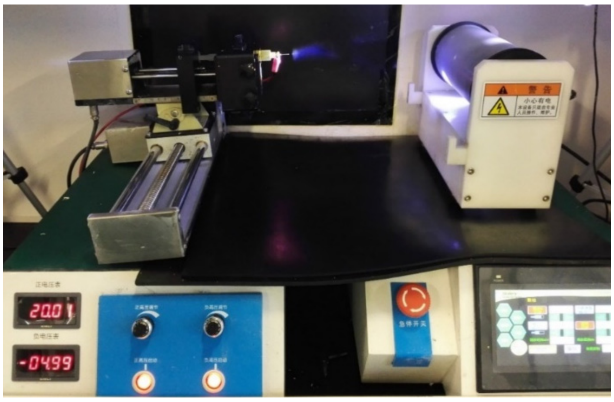

Figure S1. The electrospinning system used in this work.

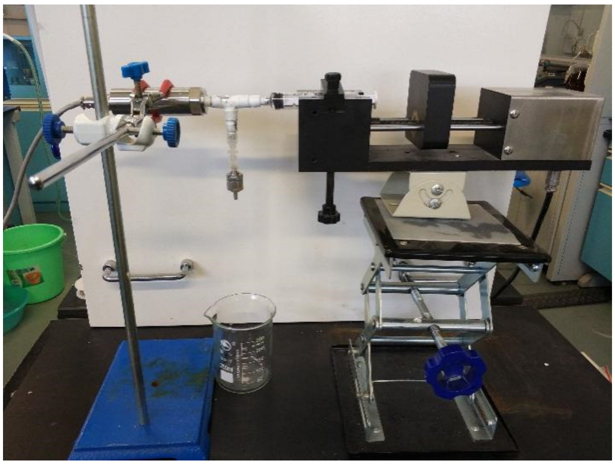

Figure S2. A LEP test unit. A syringe pump was used to generate pressure on the liquid above the test membrane by pumping the test liquid with a very low but constant flow rate (0.5 mL/min in this work).

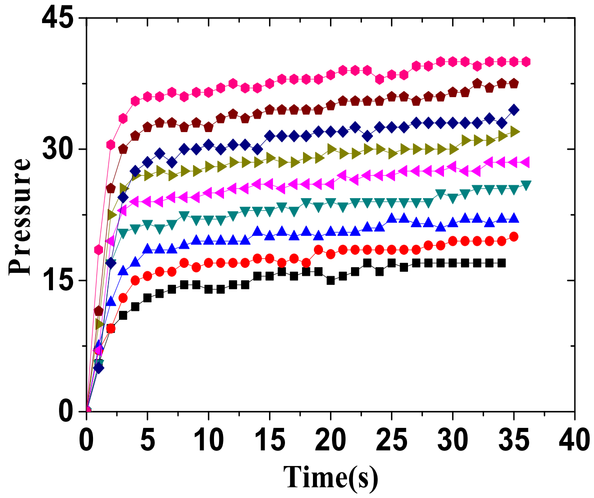

Figure S3. Schematic diagram of pressure variation under several cycles of compression with the same flow rate. The pressure increased after each cycle. (nonwoven membrane with the fiber diameter of 200 nm).
